# Severe Irritant Contact Dermatitis Causing Skin Ulceration Secondary to a Testosterone Patch

**DOI:** 10.1100/tsw.2009.45

**Published:** 2009-05-20

**Authors:** Nathan Lawrentschuk, Neil Fleshner

**Affiliations:** Division of Urology, University of Toronto, Canada

**Keywords:** testosterone, androgen, cutaneous administration, ulcer, dermatitis, hypogonadism, urology

## Abstract

Testosterone replacement has undergone somewhat of a revolution in the past decade with the introduction of topical administration techniques, including patches and gels, as well as an increasing interest in the treatment of older men with low testosterone levels for what is now termed andropause. Increasingly, testosterone replacement therapy is being individually tailored. Side effects to skin patches have been reported with irritant contact dermatitis being the most common. However, ulceration has previously not been reported. Herein, we present a case that highlights testosterone transdermal therapies, their potential side effects and management strategies, and broadens our knowledge as we approach an era where these types of treatments are likely to be more common.

## INTRODUCTION

Testosterone replacement has undergone somewhat of a revolution in the past decade with the introduction of topical administration techniques, including patches and gels, as well as an increasing interest in the treatment of older men with low testosterone levels for what is now termed andropause. This case report highlights the skin side effects of patches and provides a pathway of management for such events.

## CASE REPORT

A 35-year-old male with a history of bilateral stage I seminoma, who had been on testosterone replacement for 8 years since his second orchiectomy, presented to his urologist with a skin blister and ulcer on his left upper thigh at a site where he had been using a 2.5-mg testosterone patch (Fig. 1). The patch had been in place for the normal 24 h, but had become painful near the end of that period and so was removed. The blistering and ulceration occurred at the site of delivery of the testosterone (Fig. 2) rather than at the adhesive portion of the patch, which had left only mild erythema. At the time, he was using four patches per 24 h because the larger 5-mg patches were not available. He had previously used

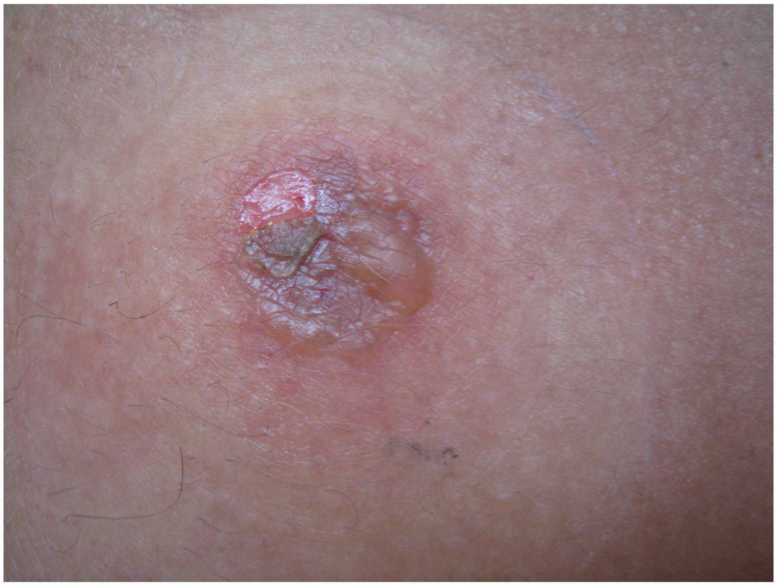

**FIGURE 1**. Blistering and ulceration secondary to irritative contact between the skin and the delivery mechanism of the drug on the patch. The adhesive at the edge of the spherical patch may be made out in the upper part of the photo

0.5% corticosteroid beneath the delivery portion of the patch with no serious reactions other than erythema, but had ceased this practice some weeks prior. The other patch sites showed no irritation. The wound was dressed after consulting dermatology and the patches rotated to other areas of skin, with the application of corticosteroid cream again to the delivery mechanism part of the patch. No other reactions were recorded, but the ulceration did take 6 weeks to heal properly with regular dressings (Fig. 3).

## DISCUSSION

Testosterone replacement has always been indicated in young hypogonadal men, but increasing yet controversial literature is emerging concerning its role in treating andropause in the aging male (symptoms including reduced muscle strength and sex drive, lethargy, mental deterioration)[1]. Increasingly, testosterone replacement therapy is being tailored to the patient because it is available in many forms, including oral, implants, intramuscular depot, and transdermal (patches and gels)[2]. Testosterone patches and gels are now extremely common and tend to mimic the natural diurnal rhythm of testosterone levels in the male[2].

One of the initial drawbacks of testosterone patches (Testoderm®, Androderm®), despite their physiological serum testosterone levels and what probably led to its slow uptake, was the recognition that the penetration-enhancing substances that had to be added to the testosterone often caused mild skin irritation[3]. Such events were generally minimal, with erythema and itchiness as main symptoms (Fig. 4)[4,5]. Nevertheless, testosterone patches are still available and used, and this is the first report of severe irritant contact dermatitis causing skin ulceration secondary to such a patch.

Irritant contact dermatitis has been defined as a nonimmunological, nonspecific reaction of the skin to an irritant. It is now recognized that this definition is too simplistic and that such reactions involve a combination of endogenous and exogenous factors that trigger a pathophysiological cascade of skin barrier disruption, cellular damage to the keratinocyte membrane, and proinflammatory mediator release, resulting in a clinical presentation[6]. Thus, ulceration may result as in this case.

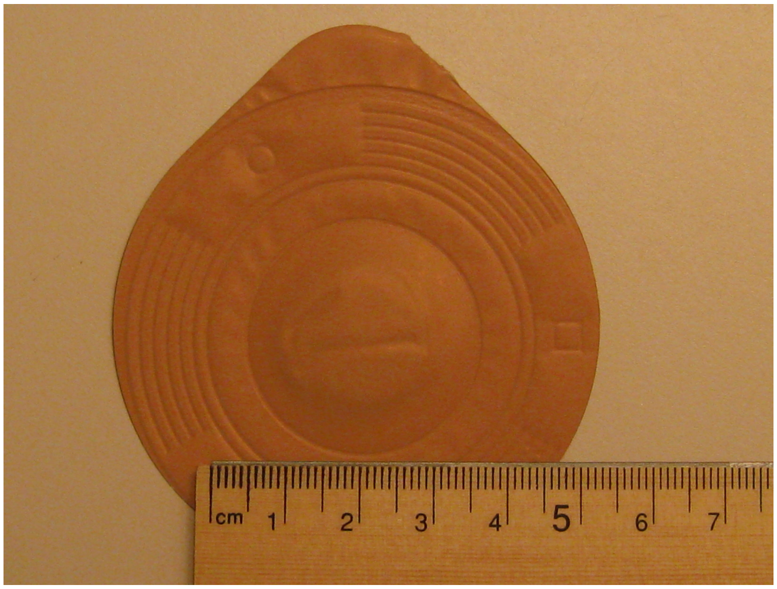

**A**

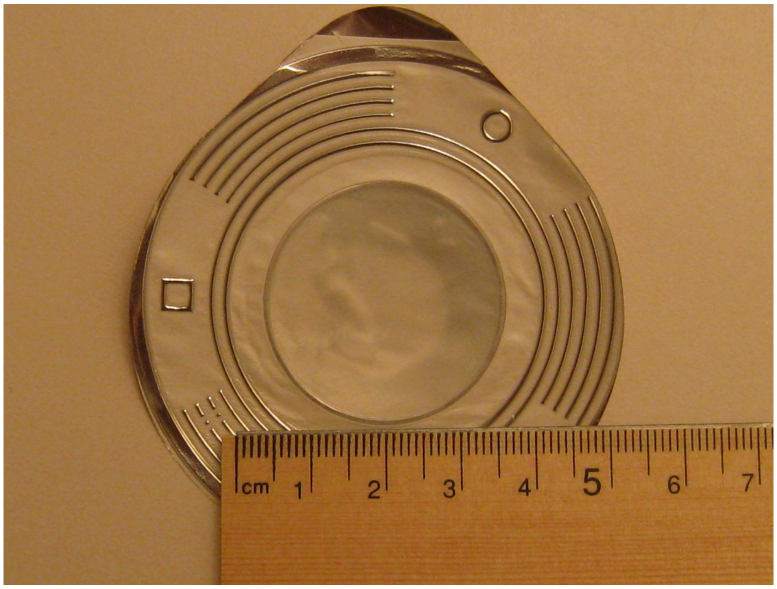

**B**

**FIGURE 2.** (A) Testosterone patch demonstrating the surface in contact with the skin with the delivery mechanism being the central area and the remainder being adhesive. (B) The reverse side of the patch is shown.

In recent updated guidelines for the management of contact dermatitis[7], the principles of treatment are avoidance, protection, and substitution of the causative agent, with a role for topical corticosteroids, soap substitutes, and emollients being widely accepted as the treatment of established contact dermatitis. For treatment of ulcers, the first clinical decision to be made is whether to repair the wound (primary closure, flaps, and graft) or to allow it to heal by second intention. Both primary- and secondary-intention wounds are aided by occlusive dressings and adjutants[8].

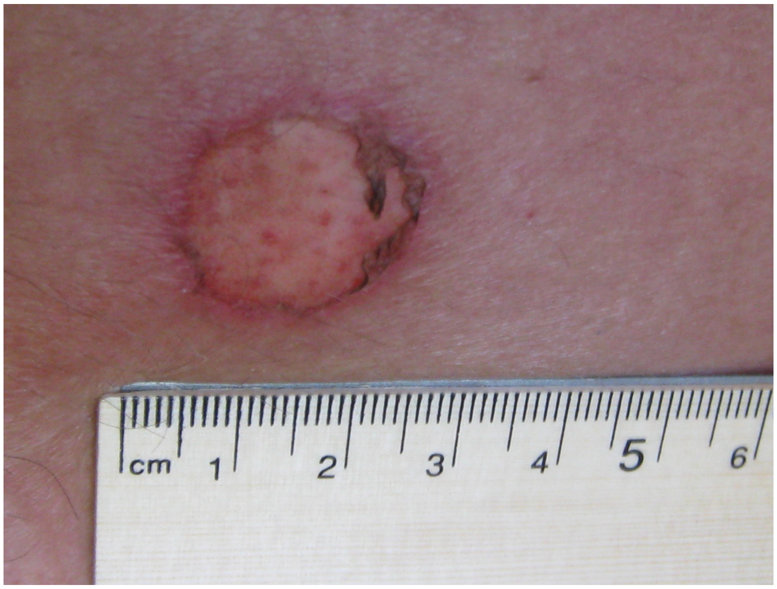

A

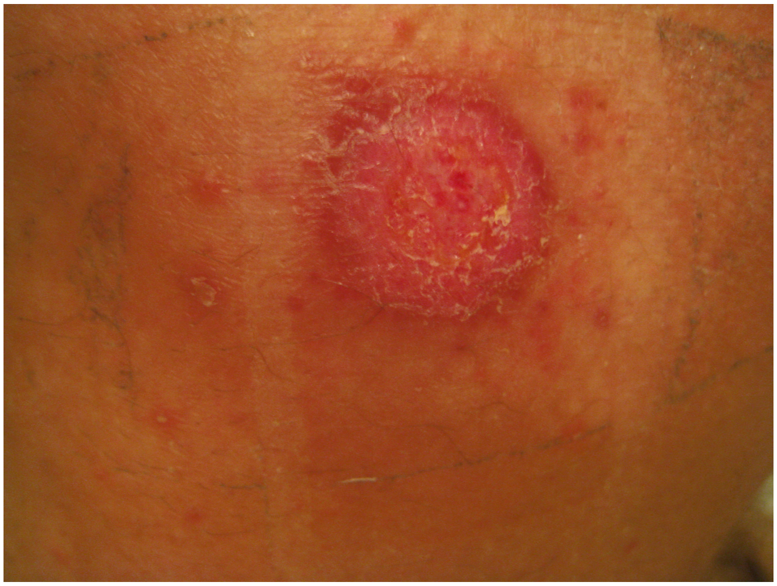

B

**FIGURE 3.** (A) The ulcer from Fig. 1 some days later, with the outer epidermis now absent with some contraction and healing at the edges; (B) 3 weeks after patch application with some general erythema and granulation tissue covering the skin ulcer.

Specifically for testosterone patches, the best treatment is recognition and discontinuation of the medication if possible and, in some instances, use of topical corticosteroids for a short period in order to relieve symptoms. However, in the case of testosterone patches, pretreatment with the corticosteroid does not reduce the bioavailability of testosterone, but decreases the severity of skin irritation and is an acceptable option. Further, irritation may be reduced or prevented by rotation of the patch application site[9].

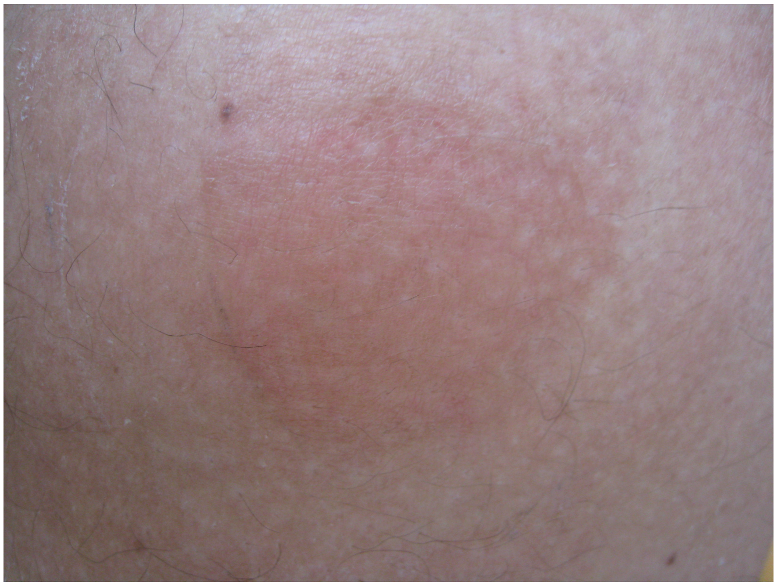

**FIGURE 4.** Erythema typical of a contact irritative dermatitis reaction secondary to the delivery mechanism of a testosterone patch.

In contrast to irritant contact dermatitis, which is by far the most common reaction, there have only been two reports of contact allergy to testosterone within a testosterone patch[4,5]. Allergic contact dermatitis to the adhesive[10], to hydroxypropylcellulose in the drug delivery reservoir[11], and to ethanol in the reservoir[12] have rarely been described as have reactions to other components in the delivery system[3]. Contact allergens include adhesives, various excipients of the patches, and the active drug molecule, especially clonidine[13].

The other transdermal mechanism of delivery is with a gel preparation (Androtop® gel, Testim®, Testogel®). Instead of having a drug delivery reservoir, the testosterone 1% gel is applied in the morning to the upper arms, shoulders, or abdomen and is left there for about 5 min to evaporate[2]. It then enters the skin, forming a physiological reservoir with slow release. Thus far, only mild erythema has been described with testosterone gel preparations that are rubbed into the skin, but not the other reactions associated with the patches[1]. If severe erythema occurs, sequential rotation of skin areas and rare discontinuation are required, with corticosteroid pretreatment not reported.

In some cases, patches may be avoided in favor of gels for irritative reasons. If a true skin allergy to testosterone develops, oral or depot administration of the same drug is generally tolerated in patients sensitized to it transdermally[13]. Oral agents remain less popular because of their poor bioavailability. Newer depot injections rather than implants have good efficacy and alleviate the need for daily medication[2]. It is important to stress that patients on testosterone replacement need regular monitoring of levels and for side effects, and, in the case of older men, PSA levels and the exclusion of prostate cancer need to be considered.

Another transdermal delivery system becoming popular in urology is an antimuscarinic treatment for overactive bladder (e.g., oxybutynin). At present, pruritus occurs in up to 15% of patients with erythema in a smaller number[14,15]. Again, topical steroids may be used[15] and there are no reported events of ulceration or severe skin reaction in the literature. Estrogen and progesterone patches also cause similar reactions to testosterone, but are rarely severe[16,17].

In summary, testosterone replacement has many options, with transdermal methods providing the most physiologic mechanisms. However, patches do have known irritative side effects and rarely may ulcerate, as in this reported case. Usually, such reactions can be avoided by rotation and pretreatment with corticosteroid cream. If this fails, gel is available and other alternatives exist when the transdermal method fails because of reaction or inconvenience. Certainly with the treatment of andropause gaining popularity, our familiarity with such agents will need to expand.
